# Geographical distribution and space–time clustering of human illnesses with major *Salmonella* serotypes in Florida, USA, 2017–2018

**DOI:** 10.1017/S0950268822001558

**Published:** 2022-10-31

**Authors:** Xiaolong Li, Nitya Singh, Arie H. Havelaar, Jason K. Blackburn

**Affiliations:** 1 Department of Environmental and Global Health, College of Public Health and Health Professions, University of Florida, Gainesville, FL, USA; 2 Emerging Pathogens Institute, University of Florida, Gainesville, FL, USA; 3 Animal Sciences Department and Food Systems Institute, University of Florida, Gainesville, FL, USA; 4 Department of Geography, Spatial Epidemiology & Ecology Research Laboratory, University of Florida, Gainesville, FL, USA

**Keywords:** Florida, *Salmonella*, serotype, spatial epidemiology

## Abstract

Nontyphoidal salmonellosis is the leading reported foodborne illness in Florida. Although the diversity of *Salmonella* serotypes circulating in Florida has been identified, the geographical characteristics of the major serotypes are poorly described. Here we examined the geospatial patterns of 803 whole-genome sequenced *Salmonella* isolates within seven major serotypes (Enteritidis, Newport, Javiana, Sandiego, Braenderup, Typhimurium and I 4,[5],12:i:-) with the metadata obtained from Florida Department of Health during 2017–2018. Geographically, the distribution of incidence rates varied distinctively between serotypes. Illnesses with Enteritidis and Newport serotypes were widespread in Florida. The incidence rate for Javiana was relatively higher in the north compared to the south. Typhimurium was concentrated in the northwest, while I 4,[5],12:i:-, the monophasic variant of Typhimurium was limited to the south. We also evaluated space–time clustering of isolates at the zip code level using scan statistic models. Space–time clusters were detected for each major serotype during 2017–2018. The multinomial scan statistic found the risk of illness with Javiana was higher in the north and southwest in the fall of 2017 compared to other major serotypes. This serotype-specific clustering analysis will assist in further unpacking the associations between distinct reservoirs and illnesses with major serotypes in Florida.

## Introduction

Nontyphoidal salmonellosis is a common foodborne illness caused by *Salmonella* bacteria. Typical symptoms include diarrhoea, abdominal cramps, vomiting and fever [1]. People contract *Salmonella* primarily through the consumption of contaminated foods or water; other pathways include direct or indirect contact with food animals, companion animals or their contaminated environments [2, 3]. As the second leading cause of reported foodborne illness in the United States (USA), salmonellosis poses a major disease burden to public health, and the Centers for Disease Control and Prevention (CDC) estimated that there are about 1.2 million illnesses, 23 000 hospitalisations and 450 deaths associated with salmonellosis occurring annually in the USA [4].

The state of Florida has consistently been one of the states in the USA with the highest incidence rates of salmonellosis which is the leading reported foodborne illness in Florida [5]. On average, approximately 5000–6000 *Salmonella* cases are reported each year. The incidence rate of salmonellosis ranged from 27.8 to 36 cases per 100 000 person-years during 2009–2018 in Florida, nearly twice as high as the national average (15.2–18.6 cases per 100 000 person-years) in the same time frame [6].

More than 2500 *Salmonella* serotypes have been identified, with a great diversity of reservoirs and transmission pathways being associated with them [7]. For example, humans are considered to be the only host species of serotype Typhi; while the primary reservoir for serotypes Choleraesuis and Dublin is known as pigs and cattle, respectively. Some serotypes are mainly transmitted by food, such as the serotypes Saintpaul, Heidelberg and Berta, but others such as Bareilly and Mississippi appear to be less linked to food pathways [8]. The five most common serotypes identified through the FoodNet surveillance system in the USA are Enteritidis, Typhimurium, Newport, Javiana and I 4,[5],12:i:-, a monophasic variant of Typhimurium [9], while the major serotypes occurring in Florida are Enteritidis, Newport, Javiana, Sandiego and Braenderup [6]. In our previous work, we conducted a phylogenetic analysis of *Salmonella* isolates from five dominant serotypes in Florida and identified the geographical distribution of clusters of genetically related isolates using core genome multi-locus sequence typing (cgMLST), a subtyping approach that provides high resolution to distinguish isolates within the same serotype [10]. Eight clusters of genetically related isolates from four serotypes (Enteritidis, Newport, I 4,[5],12:i:- and Bareilly) involved more than 10 isolates and were investigated. There were isolates distributed in adjacent counties of specific regions within each cluster; however, overall, the isolates involved were separated by considerable distances. The results provided some insight into the geographical distribution of *Salmonella* isolates at a finer level within serotypes, but we still lacked the overall picture of the geospatial patterns of isolates at the serotype level, given the rich diversity of *Salmonella* serotypes in Florida [10]. In this paper, we aimed to determine the geographical distribution of illnesses with major *Salmonella* serotypes in Florida and to identify the clustering of high rates on the landscape spatially and temporally by employing the scan statistics [11].

The scan statistics are a set of popular statistical approaches for detection of spatial and spatio-temporal clusters in geographical disease surveillance as well as other fields, such as criminology and entomology [11]. It can be further divided into spatial, space–time and temporal scan statistics which are used to determine the statistical significance of clusters in space and/or time through a hypothesis-testing process with the null hypothesis that all observations are randomly distributed and follow the same distribution [12]. Under the alternative hypothesis, one cluster location is identified if the number of cases within the cluster exceeds the expected under the null model. The advantage of this approach is that prior knowledge of the locations, time periods and size of clusters is not required. Scan statistics are commonly used geospatial tools in studies of foodborne pathogens and related illnesses. Such tools have been successfully applied in detecting spatial and/or temporal clusters of human illnesses caused by varying *Salmonella* serotypes in Europe and North Americas [13–17]. Their strength in capturing spatial and space–time clusters combined with high-resolution molecular subtyping data expands our ability to assist foodborne disease surveillance and outbreak detection [17].

## Methods

### Data sources

Salmonellosis is a reportable disease in Florida, and laboratories in the state are required to send isolates or specimens of *Salmonella* spp. to the Florida Bureau of Public Health Laboratories (BPHL) for confirmation or additional characterisation [18]. The BPHL started whole-genome sequencing (WGS) isolates submitted by local laboratories in 2017. As described elsewhere [6], metadata of sequenced isolates between 2017 and 2018 were obtained from the BPHL Bionumerics database, and related epidemiological and demographic data including age, sex, isolation date, ethnicity and five-digit zip code were obtained from the Florida Department of Health Merlin database. A total of 2507 complete records, 1353 isolates from 2017 and 1154 isolates from 2018 were available from both databases.

### Serotype prediction

Serotype prediction was performed in the EnteroBase online server (https://enterobase.warwick.ac.uk/) [19] as described in [6]. We included the top five *Salmonella* serotypes in Florida, plus two additional serotypes, Typhimurium and I 4,[5],12:i:- (a monophasic variant of Typhimurium), that are on the list of top five serotypes across the whole USA (Table 1).

**Table 1.** Major *Salmonella* serotypes in the state of Florida included in this study

Serotype	Number reported	Proportion (%)	Incidence rate/100 000
Enteritidis	185	11	0.90
Newport	163	9.5	0.79
Javiana	123	7.2	0.60
Sandiego	123	7.2	0.60
Braenderup	91	5.3	0.44
Typhimurium	83	4.9	0.40
I 4,[5],12:i:-	35	2.1	0.17

### Disease mapping

To represent the overall distribution of human salmonellosis with these major serotypes reported in Florida between 2017 and 2018, cases of these 2 years were aggregated to county level for disease mapping. As per ethical requirements for the purpose of visualisation, counties with a population less than 20 000 were merged with other counties based on contiguous and comparable geographical locations (coastal or inland) and median household income, a total of 58 merged counties (collapsed from 67 counties) were used in this study [20]. The raw incidence rate of *Salmonella* cases at the county level was calculated through dividing the 2-year total number of serotype-specific *Salmonella* cases per county by two times of county-level population estimate in 2017 (assuming the population at the county level did not change too much between 2 years).

As the highly varying population among geographic units may affect the precision of rate estimate and yield variance instability of incidence rate, the spatial empirical Bayes method was used to smooth these rates and reduce the degree of instability in rates [21]. Based on a shrinkage principle, the rate estimate based on a larger population at risk has small variance and will be marginally shrunk; whereas the rate with large variance (based on smaller population) will have a large shrinkage towards the regional mean of spatial units defined by a weights matrix. The spatially smoothed incidence rate of salmonellosis for each serotype at the county level was calculated in GeoDa version 1.20.0 software (https://geodacenter.github.io/download.html) with a weights matrix created using the *k*-nearest neighbours algorithm (*k* = 5). These incidence rates were then choropleth mapped in ArcMap 10.8 (ESRI, Redlands, CA, USA). Annual estimates of county resident population in Florida were obtained from the FloridaHealthCharts (http://www.flhealthcharts.com/, accessed on 30 November 2020).

### Scan statistical analysis

#### Retrospective purely spatial scan statistic with Poisson model

The spatial clustering of illnesses with major *Salmonella* serotypes in Florida during the study period was identified using the purely spatial Poisson model in SaTScan version 9.6 (https://www.satscan.org/). The coordinates (latitude/longitude pairs) for centroids of zip code areas in Florida were calculated in ArcMap 10.8. The 2018 population data for each zip code area were obtained from the US Census (accessed at https://data.census.gov/cedsci/ on 30 November 2020) as the population file. As our previous study indicated that the range of most putative foodborne illness events in Florida was limited to the vicinity of where the consumers live [20], we ran the purely spatial Poisson model with the maximum cluster size being up to 25% of the population at risk. Only clusters with high rates were identified, and the significance level was set at *α* = 0.05. Purely spatial Poisson models were built for each serotype separately to capture the serotype-specific spatial clusters.

#### Retrospective space–time scan statistic with Poisson model

To test for space–time clustering of salmonellosis data points at the zip code level during 2017–2018, we first built separate space–time models for these seven serotypes by employing a retrospective discrete Poisson model in SaTScan. For the temporal component in the model, isolation date of *Salmonella* isolates was used for the temporal scanning with month as the smallest temporal unit. Since the salmonellosis cases reported in Florida demonstrated a strong seasonality [6], we selected a 3-month period as the maximum temporal window to detect clusters in time. The other parameters were the same as the purely spatial Poisson model. For all spatial and space–time scan statistics, we considered primary and secondary clusters so long as they were statistically significant.

#### Multinomial scan statistic model

In addition to investigating the clustering of illnesses with separate serotypes, we also considered all seven serotypes simultaneously to examine the illness pattern in space and time across the study area with a space–time multinomial scan statistic model [22]. Serotype was the categorical variable used in the multinomial model, and this approach compared all possible groupings of the serotypes to detect clustering within which a grouping was significantly different from the rest of study area. The settings of significance level, maximum spatial window and the maximum temporal window were the same as for the retrospective discrete Poisson model.

## Results

### Disease mapping

A total of 803 salmonellosis cases with these seven major serotypes were included in the analysis, which accounted for 47% of total sequenced isolates with available serotype information. A large proportion of laboratory-sequenced isolates came from the age group under-5-years. For ethnicity, although non-Hispanic cases were predominant, nearly 30% of the sequenced isolates were reported as Hispanic or Latino. Detailed demographics of the sequenced isolates can be found elsewhere [6]. Illnesses with serotype Enteritidis were most common (11%), while illnesses with I 4,[5],12:i:- were least common (2.1%) (Table 1).

**Fig. 1.**
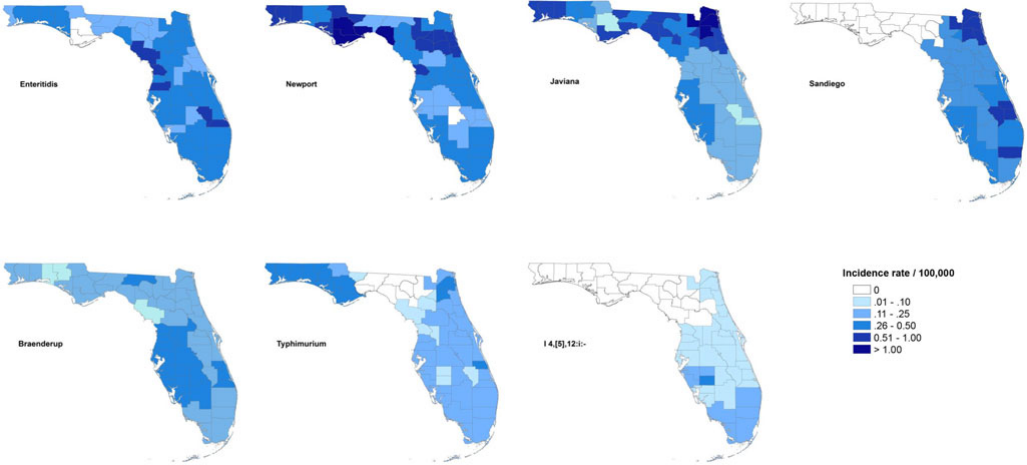
Geographical distribution of spatially smoothed incidence rates of major *Salmonella* serotypes in Florida, 2017–2018.

The maps of raw salmonellosis incidence rate at the county level can be found in the Supplementary material (Fig. S1). After spatial smoothing, the incidence rates of illnesses with major serotypes in counties of Florida ranged from 0 to 0.58 per 100 000 person-years for Enteritidis, 0 to 2.12 per 100 000 person-years for Newport, 0.06 to 1.10 per 100 000 person-years for Javiana, 0 to 0.84 per 100 000 person-years for Sandiego, 0.06 to 0.39 per 100 000 person-years for Braenderup, 0 to 0.49 per 100 000 person-years for Typhimurium and 0 to 0.26 per 100 000 person-years for I 4,[5],12:i:- (Fig. 1).

The distribution of incidence rates at the county level varied among serotypes, and some demonstrated obvious patterns geographically. Illnesses with Enteritidis and Newport were distributed across the state. The incidence rate of illness with Javiana was much higher in northeast and northwest Florida than in the central and south, and illness from Sandiego was concentrated on the east coast. Coastal areas in west central and south Florida had a relatively higher incidence rate of illness with Braenderup, while counties in the west of the Florida Panhandle showed a higher incidence rate of illness with Typhimurium. For serotype I 4,[5],12:i:-, counties with higher incidence rate were primarily in the south, especially the southeast.

### Scan statistics

#### Purely spatial scan statistic with Poisson model

**Fig. 2.**
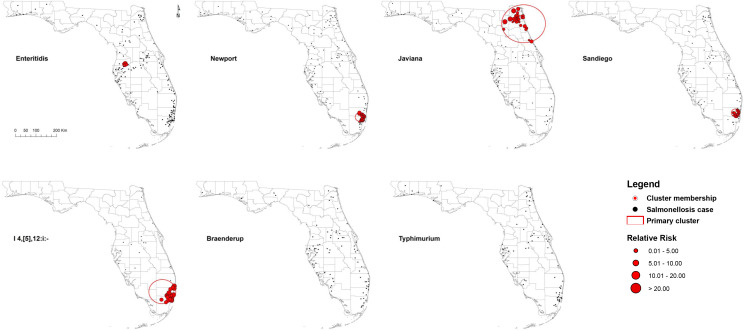
Purely spatial clusters of illnesses with major *Salmonella* serotypes in Florida, 2017–2018. Red dots represent salmonellosis cases within a primary cluster. No purely spatial clusters for Braenderup and Typhimurium serotypes were detected.

Five high-rate spatial clusters were detected for serotypes Enteritidis, Newport, Javiana, Sandiego and I 4,[5],12:i:-. No clusters of Braenderup and Typhimurium were identified throughout the 2 years (Fig. 2). Variations in local relative risk (RR) for zip code areas included in each cluster were provided in the Supplementary material.

One Enteritidis cluster was in central Florida with seven cases involved. The risk of getting infected with Enteritidis within this cluster was 9.35 times higher than that outside of the cluster. Three clusters for I 4,[5],12:i:-, Newport and Sandiego, respectively, were distributed in the coastal counties of southeast Florida, especially Miami-Dade and Broward counties. The size and RR of these clusters ranged from 20 to 31 cases and 2.62–5.43, respectively. In addition, a relatively large-scale cluster of 39 Javiana cases was detected in northeast Florida with an RR of 4.97.

#### Retrospective space–time Poisson model

**Fig. 3.**
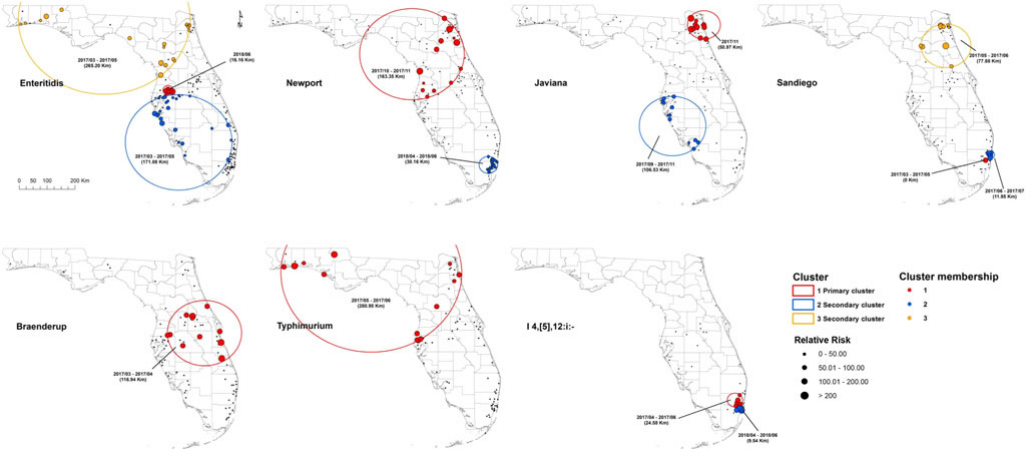
Space–time clusters of illnesses with major *Salmonella* serotypes in Florida, 2017–2018. A space–time retrospective Poisson model was used to detect clusters with high rate. The number in the brackets represents the radius of the corresponding cluster.

According to the space–time scan statistical analyses on illnesses with major *Salmonella* serotypes at the zip code level, one or more high-rate, space–time clusters were detected for each serotype (Fig. 3). Eleven statistically significant primary/secondary clusters were distributed in 2017 and three in 2018 (Table 2).

**Table 2.** Space–time clusters of illnesses with major *Salmonella* serotypes in Florida, 2017–2018

Serotype	Cluster	Time frame	Radius/km	Observed nos.	Expected nos.	O/E	RR	*P*-value
Enteritidis	C1	2018/6	16.16	8	0.08	98.67	103.44	<0.001
	C2	2017/3-2017/5	171.08	28	5.21	5.37	6.23	<0.001
	C3	2017/3-2017/5	265.20	20	3.06	6.54	7.27	<0.001
Newport	C1	2017/10–2017/11	163.35	18	3.14	5.74	6.34	<0.001
	C2	2018/4–2018/6	30.16	16	2.89	5.54	6.04	0.005
Javiana	C1	2017/11	50.97	11	0.30	36.57	40.19	<0.001
	C2	2017/9–2017/11	106.53	15	2.55	5.88	6.58	0.012
Sandiego	C1	2017/3–2017/5	0	5	0.05	103.40	107.81	<0.001
	C2	2017/6–2017/7	11.95	7	0.29	24.50	25.95	0.003
	C3	2017/5–2017/6	77.88	10	0.94	10.59	11.46	0.007
Braenderup	C1	2017/3–2017/4	116.94	12	1.75	6.84	7.77	0.015
Typhimurium	C1	2017/5–2017/6	280.95	13	1.53	8.49	10.01	<0.001
I 4,[5],12:i:-	C1	2017/4–2017/6	24.58	6	0.28	21.07	25.22	0.013
	C2	2018/4–2018/6	9.54	5	0.17	29.34	34.07	0.028

One primary cluster and two secondary clusters were identified for serotype Enteritidis. The primary cluster was relatively small with a radius of 16.16 km, reflecting a concentration of cases. Eight isolates obtained in June 2018 were included, and the RR was as high as 103.44. The secondary clusters both occurred between March 2017 and May 2017; one was found in the south and the other covered the northwest and northeast region of Florida.

The primary cluster of Newport isolates covered northeast and central Florida with a radius of 163.35 km. Eighteen isolates were involved during the period of October 2017–November 2017, and the RR was 6.34. A small secondary cluster involving 16 isolates was identified in the southeast coastal area between April 2018 and June 2018, with an RR of 6.04.

For serotype Javiana, the primary cluster including 11 isolates was identified in the northeast in November 2017. Additionally, 18 isolates were clustered in the southwestern part of Florida during September 2017–November 2017 as the secondary cluster. The RR was 40.19 for the primary cluster and 6.58 for the secondary cluster.

For serotype Sandiego, five isolates located in a single zip code area of southeast Florida constituted the primary cluster between March 2017 and May 2017, with a very high RR of 107.81. One secondary cluster was close to the primary cluster and included seven isolates (RR = 25.95) between June 2017 and July 2017. Another secondary cluster was in the northeast part of Florida and included 10 isolates (RR = 11.46) between May 2017 and June 2017.

The primary cluster of illnesses with Braenderup was in central Florida between March 2017 and April 2017. Twelve salmonellosis cases were involved, and the RR of getting infected with Braenderup within this cluster was 7.7 times as high as in the areas outside of the cluster.

For serotype Typhimurium, the primary cluster included 13 isolates in northwest, northeast and central Florida during May 2017–June 2017.

The primary and secondary clusters of illnesses with I 4,[5],12:i:- were adjacent in the southeast coastal areas. The primary cluster included six isolates obtained between April and June of 2017, and five isolates were in the secondary cluster between April and June of 2018. The RR was 25.22 for the primary cluster and 34.07 for the secondary cluster.

#### Multinomial scan statistic model

**Fig. 4.**
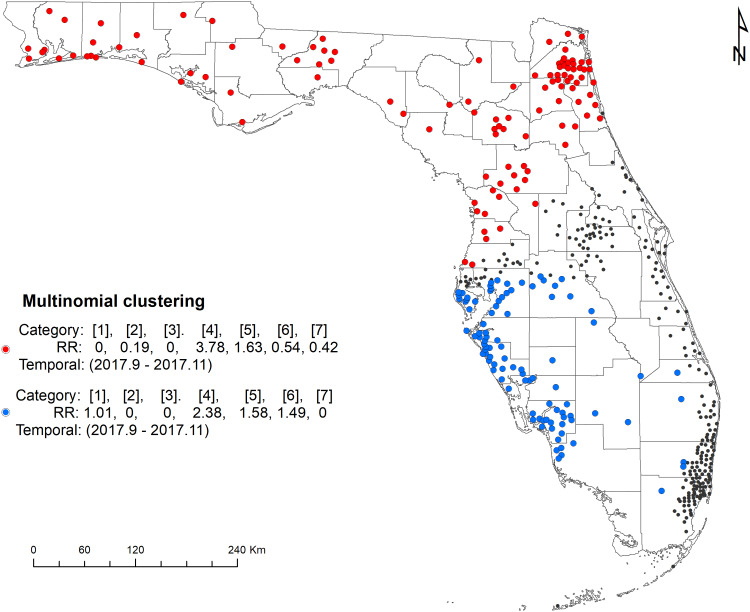
Space–time clusters of illnesses with major *Salmonella* serotypes identified by a multinomial scan statistic model. Red dots represent the primary cluster, and blue dots the secondary cluster. Black dots are salmonellosis cases not belonging to any cluster. Categories in brackets refer to the serotype of *Salmonella* isolates: 1 = Braenderup, 2 = Enteritidis, 3 = I 4,[5],12:i:-, 4 = Javiana, 5 = Newport, 6 = Sandiego and 7 = Typhimurium. RR indicates whether the observed number of isolates for one category within the cluster is greater than the expected number (RR > 1).

The space–time multinomial scan statistical analysis with a 3-month maximum temporal window detected two space–time clusters (Fig. 4). The primary cluster involved a large part of north Florida, covering the Florida Panhandle and part of northeast and central Florida from September 2017 to November 2017. Among the seven serotypes, Javiana and Newport had an RR greater than 1 and all the other five had an RR less than 1 (Table 3). The secondary cluster was detected in the south with a radius of 169.22 km. It also occurred between September 2017 and November 2017. The RRs for Braenderup, Javiana, Newport and Sandiego were greater than 1 with Javiana having the greatest value. The other three serotypes had an RR of 0.

**Table 3.** Space–time clusters of illnesses with major *Salmonella* serotypes identified by a multinomial scan statistic model in Florida, 2017–2018

Cluster	Time frame		Braenderup	Enteritidis	I 4,[5],12:i:-	Javiana	Newport	Sandiego	Typhimurium	*P*-value
C1	2017/9–2017/11	Observed	0	2	0	23	15	4	2	0.001
		Expected	5.19	10.26	2.09	7.10	9.55	7.22	4.59	
		O/E	0	0.19	0	3.24	1.57	0.55	0.44	
		RR	0	0.19	0	3.78	1.63	0.54	0.42	
C2	2017/9–2017/11	Observed	5	0	0	15	14	10	0	0.001
		Expected	4.96	9.82	2.00	6.79	9.13	6.91	4.39	
		O/E	1.01	0	0	2.21	1.53	1.45	0	
		RR	1.01	0	0	2.38	1.58	1.49	0	

## Discussion

A variety of *Salmonella* serotypes contribute to salmonellosis cases in Florida, and there is little information on possible sources of exposure. We conducted serotype-specific spatial and spatiotemporal analyses using WGS *Salmonella* isolates obtained between 2017 and 2018 to determine the distribution and test for spatiotemporal clustering of illnesses of seven major serotypes in Florida. To account for the instability in the incidence rate caused by the small population in some geographic units [23], spatial Bayesian smoothing was employed in this study to obtain a more robust picture of the overall spatial pattern of areas with high- and low- incidence rates.

The distribution of areas with a high-incidence rate varied distinctively between serotypes. Serotypes Enteritidis and Newport were the most common *Salmonella* serotypes in both Florida and the USA. Illnesses with these two serotypes were widespread covering almost all counties of Florida. The areas of high-incidence rate for Enteritidis tended to be in central and south Florida, whereas the northwestern and northeastern parts had more areas with high-incidence rates than the south for serotype Newport.

Serotype Sandiego did not appear in the top 20 list nationally but was ranked fourth in Florida. Illnesses with Sandiego were distributed across Florida except for the Panhandle area. Reptiles including turtles, lizards and iguanas have been recognised as reservoirs of *Salmonella* for decades [24–26], and serotype Sandiego was reported previously in several multistate outbreaks of human illness associated with small pet turtle exposure in the USA [27–29]. Whether the distribution of reptiles in Florida contributes to this specific pattern of illnesses with Sandiego deserves further investigation. In contrast, serotype I 4,[5],12:i:-, a monophasic variant of Typhimurium, was less common in Florida than nationally. This serotype has been increasingly isolated in pigs and pork products that contribute to human outbreaks worldwide [30, 31]. However, Florida is not a major state in pig farming and sales [32], which may partly explain why the incidence of serotype I 4,[5],12:i:- was relatively low in Florida.

*Salmonella* Javiana cases were limited to southern Florida initially in the 1980s, and the range gradually expanded to all of Florida as well as other states in the southeastern region of the USA afterwards [10]. Here, we found that the incidence rate of illnesses with Javiana was relatively higher in the northern parts of Florida than in the south during 2017 and 2018. Among the seven major serotypes, Javiana had the highest RR within the detected clusters covering northwest and northeast Florida and the coastal areas of southwest Florida between September and November of 2017. Previous studies have shown that both food and non-food (e.g. environmental pathway and animal contact) exposures contribute to the transmission of human illnesses with Javiana [33]. In the USA, multiple outbreaks of illness with Javiana were associated with the consumption of tomatoes, specifically Roma tomatoes [34, 35]. In one multistate outbreak affecting primarily transplant organ recipients who attended the 2002 US Transplant Games in Orlando, Florida, the investigation indicated diced Roma tomatoes were significantly associated with the illnesses with Javiana [34]. Case-control studies conducted in several southeastern states including Mississippi, Georgia and Tennessee indicated contact with amphibians or reptiles and their environments may also be considered a risk factor of illness with Javiana among humans [36, 37]. The distribution of certain amphibian species exclusively in the southeastern regions of the USA may partly contribute to the geographic pattern of human illnesses from Javiana [37]. In addition, environmental habitats, such as wetland cover, could also play a role in the transmission of Javiana by sustaining the existence of these reservoirs [38]. Whether the presence of these risk factors/reservoirs in Florida can be associated with the pattern of Javiana identified in this study would be an interesting research direction in the future.

Another interesting phenomenon was that the space–time clusters for serotypes Newport and I 4,[5],12:i:- were overlapping in the coastal area of southeast Florida from April to June 2018. It may imply a co-circulation of outbreak-related serotypes in this area. Additionally, two clusters of Sandiego cases were identified around this area in 2017, suggesting a diversity of serotypes circulating in the southeast part of Florida.

By comparing the distributions of spatial clusters and space–time clusters identified in this study, we found several seasonal patterns for these major serotypes. These patterns might be linked to the corresponding sources of exposure. The purely spatial clusters captured the hotspots of high risk of illness with major serotypes in Florida within a 2-year time span. After adding a 3-month maximum temporal window in the space–time scan statistic, we were able to identify space–time clusters located not only within but also outside of the purely spatial clusters. One or two space–time clusters were nested in all five purely spatial clusters, respectively. For example, one space–time cluster of Newport occurred between April and June of 2018 and was located within the purely spatial cluster in the southeast coastal area. Similarly, space–time clusters of I 4,[5],12:i:- occurred between April and June of 2017 and another cluster occurred during the same time frame in 2018, both inside the purely spatial cluster of I 4,[5],12:i:-. This can be interpreted as seasonal spikes within a hotspot of illnesses with specific serotypes. Another pattern was that space–time clusters occurred in areas where no purely spatial cluster was detected. There were two space–time clusters of Enteritidis with greater radius covering a large area of both north and south Florida. They both took place between March and May of 2017, indicating that, although Enteritidis cases are widespread in Florida, more cases tend to occur in spring for areas within the space–time clusters. Although no purely spatial clusters were identified for Braenderup and Typhimurium, one space–time cluster for Braenderup (March–April 2017) and one for Typhimurium (May–June 2017) were found in central and north Florida, respectively. The Braenderup cluster coincides with a multistate outbreak related to backyard chickens [39]. Meanwhile, a multistate outbreak of Typhimurium related to clinical and teaching microbiology labs was reported in the same time frame as the Typhimurium cluster [40], and this coincidence has been confirmed by WGS [10]. These results might help provide some insight into the seasonality of these two serotypes in Florida. This pattern was not observed in I 4,[5],12:i:-. A lower number of cases included in the analysis might be one possible reason. Another explanation could be the distribution of high-incidence areas was restricted to the southeastern part of Florida with a peak of April–June.

Overall, these identified clusters spanned from March through November between 2017 and 2018. However, the majority occurred in spring/early summer (March–June), which aligns with the previous finding that spring had a relatively higher richness of common *Salmonella* serotypes in the USA [41]. One Newport cluster along with all Javiana clusters took place in fall (September–November). Meanwhile, Javiana and Newport had a higher RR compared to other major serotypes during September–November in certain areas as indicated by the multinomial scan statistic, which can be explained by the similar seasonality between Javiana and Newport. According to the atlas of *Salmonella* in the USA, most Javiana and Newport cases were observed during July through October among different age groups [7, 42]. In contrast, for serotype Enteritidis, although having the highest incidence in Florida and across the USA, its seasonality was spread out throughout the year [42]. One explanation could be Javiana and Newport are attributed to more local and natural reservoirs [7], whereas Enteritidis is more likely to be linked to a common food type, especially chicken meat and eggs, transported from common sources across the USA [10].

The distribution of clusters of genetically related *Salmonella* isolates detected in our previous phylogenetic work [10] was consistent with the geographical pattern of areas with a high-incidence rate of illness from varying serotypes. The hierarchical clusters based on cgMLST-clustered isolates were based solely on their genetic relatedness, without considering the spatial and temporal components in the data. Isolates identified in the same hierarchical cluster are not necessarily close geographically and might be located hundreds of kilometres apart. This feature could be useful when capturing the trajectory of transmission of cases in outbreaks but would not be efficient for the detection of specific hotspots in space and time. The differences in the definition of clusters and in algorithms of identifying clusters utilised in these two studies make it difficult to directly compare the locations of these clusters. However, we did find several overlaps in clusters detected using both approaches. For example, one hierarchical cluster of Newport involving 34 isolates was distributed in two adjacent counties (Miami-Dade and Broward) in the southeast of Florida. Here, both purely space and space–time scan statistic models identified clusters covering these two counties as well. In the future, the combination of these two approaches might be applied in foodborne illness-related outbreak detection and tracking. Scan statistic models could be used to prompt early outbreak detection within the routine surveillance of foodborne illnesses. The New York City Department of Health and Mental Hygiene has proved its effectiveness in detecting a salmonellosis outbreak of five patients via an automated space–time analysis system using SaTScan software [43]. Following that, hierarchical clusters based on cgMLST could extend the outbreak detection by linking genetically related cases at a larger scale and tracking the potential sources of the outbreak. The application of this set of strategies is not limited to Florida or other states in the USA but will also benefit other countries or regions around the world, e.g. European countries where the burden of foodborne illnesses including salmonellosis and campylobacteriosis remains high and developed surveillance systems are already in place. The scan statistic models can be integrated into their surveillance systems for the purpose of first-pass outbreak detection, and genetic data obtained from the laboratories can be used to trace the progress of outbreaks as a further step.

Following the determination of the geographical distribution of illnesses with major *Salmonella* serotypes, several interesting findings could become our future research directions. It will be worthwhile to relate these cases to their potential sources of exposure (food *vs*. non-food) and further examine the preferred transmission pathways of different serotypes. The Florida Department of Health aims to interview all reported salmonellosis cases, with a prioritisation on those who are part of a possible outbreak or are in a sensitive situation, such as attendees or employees of childcare centres or food handlers [20]. The interview questions used for this purpose cover possible food or non-food exposures that reported cases may have experienced. Future studies could connect this information to the detected clusters and identify putative causal factors.

The results from this study will also guide us to develop hypotheses to further explore the risk behaviours related to different serotypes. Risk factors vary between *Salmonella* serotypes depending on food and non-food pathways. Some serotypes are more food-related, while others may have a higher risk of environmental exposure [7, 8]. For the environmental reservoirs, it is interesting to note the overlap of distribution of certain amphibian and reptile species with the geographical pattern of human salmonellosis cases in Florida as well as other southeastern states. In future, we may need to collect sequence data of *Salmonella* isolates from amphibians or reptiles as well as the exposure of reported human cases to these cold-blooded vertebrates.

Geographical factors such as urban/rural areas and coastal/inland areas could affect the patterns of food consumption. For example, people living in urban areas are more likely to eat at restaurants than people living in rural areas [20]. Likewise, people living in coastal areas have more opportunities to consume seafood.

Ethnicity is another factor to consider. According to the US Census Bureau population estimates in 2021, 26.8% of the Florida population were Hispanic or Latino [44]. And counties with a high percentage of Hispanic-Latino population were primarily located in the south of Florida, with Miami-Dade county having the highest percentage (69.1%). In this study, for all serotypes, 61.3% of the salmonellosis cases in Miami-Dade county were Hispanic, which is similar to the population structure in this county. We previously reported that 29.7% of sequenced *Salmonella* isolates (including all serotypes) came from this group, which was slightly higher than the percentage of 26.1 in the whole Florida population [6]. Case-control studies to assess whether ethnicity is a potential risk factor for specific serotypes of *Salmonella* in some areas of Florida could be guided by the clusters detected in this study.

There were several limitations in this study. Of all the WGS-sequenced isolates included in the analysis, isolates obtained in 2018 accounted for 29% due to the availability in the EnteroBase platform for serotype prediction, which may partly contribute to the result of fewer space–time clusters being detected in 2018. Thus, it is well worth conducting a follow-up analysis with updated isolate information from EnteroBase in the subsequent years and monitoring the prevalence of illnesses with major serotypes in Florida.

Isolation date, which may have a lag of a few days or even weeks after the date of collection, was used in the space–time clustering analysis. Although the selection of a 3-month temporal window could account for this lagging to some extent, it might be more efficient in assisting the outbreak detection if the collection date could be integrated into the scan statistic in the future.

This study confirms spatial patterns and seasonality of illnesses varied distinctively among major *Salmonella* serotypes in Florida. Notably, illnesses with Enteritidis and Newport serotypes were widespread in Florida; while Typhimurium was concentrated in the northwest, and I 4,[5],12:i:-, was limited to the south. For seasonality, Javiana and Newport had a higher RR compared to other major serotypes during September to November in certain areas. In contrast, the seasonality of Enteritidis was spread out throughout the year. These results will assist in further unpacking the associations between distinct reservoirs and illnesses with major serotypes in Florida as well as identifying potential environmental and socioeconomic risk factors for salmonellosis. This will benefit the prevention and control of foodborne illnesses like salmonellosis and improve food safety not only in Florida but also in other regions having similar settings.

## Data Availability

The datasets presented in this study can be found online at: https://dataverse.harvard.edu/dataverse/Salmonella_FL.

## Supplementary material

For supplementary material accompanying this paper visit http://doi.org/10.1017/S0950268822001558. click here to view supplementary material
