# Supplementary material for: Geographical distribution and space–time clustering of human illnesses with major Salmonella serotypes in Florida, USA, 2017–2018
Source: Epidemiol Infect. 2022 Oct 31;150:e175. doi: 10.1017/S0950268822001558 (PMC9980922; doi:10.1017/S0950268822001558)
Supplement: Supplementary file 1 [file S0950268822001558sup001.docx]

Supplementary Material

Supplementary Figure S1. Raw incidence rates of illnesses with major *Salmonella* serotypes in Florida, 2017-2018


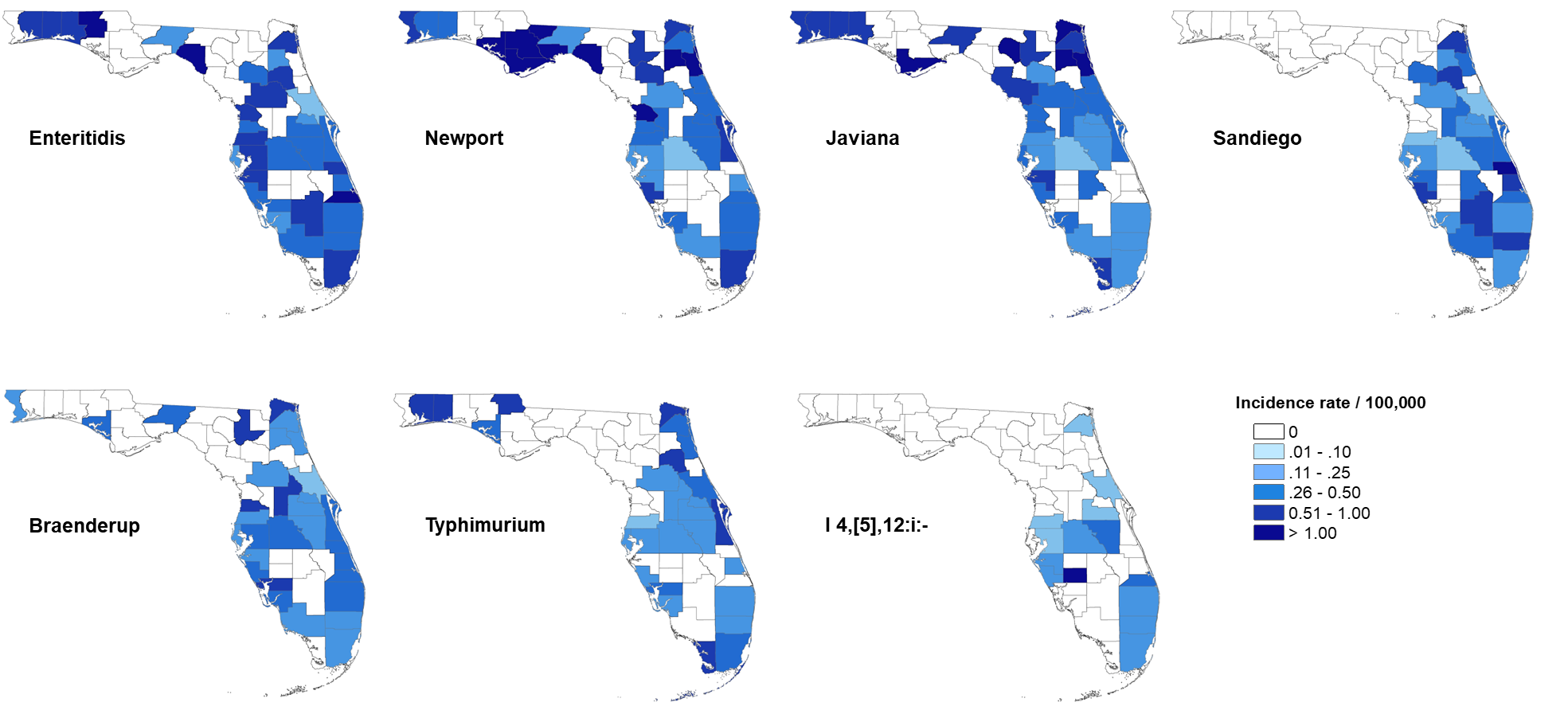


Supplementary Table S1. Local relative risks of zip code areas within the detected significant space only clusters of illnesses with major *Salmonella* serotypes in Florida, 2017-2018

| Serotype | Location ID | Cluster | Local obs # | Local exp # | Local O/E | Local RR |
| --- | --- | --- | --- | --- | --- | --- |
| Enteritidis | 574 | 1 | 2 | 0.205 | 9.778 | 9.881 |
|  | 576 | 1 | 3 | 0.139 | 21.523 | 21.888 |
|  | 575 | 1 | 1 | 0.250 | 3.995 | 4.012 |
|  | 572 | 1 | 1 | 0.183 | 5.464 | 5.491 |
| Newport | 212 | 1 | 1 | 0.533 | 1.876 | 1.881 |
|  | 217 | 1 | 1 | 0.488 | 2.049 | 2.056 |
|  | 179 | 1 | 3 | 0.596 | 5.032 | 5.109 |
|  | 218 | 1 | 1 | 0.220 | 4.539 | 4.561 |
|  | 220 | 1 | 1 | 0.360 | 2.778 | 2.789 |
|  | 219 | 1 | 1 | 0.246 | 4.065 | 4.084 |
|  | 186 | 1 | 1 | 0.196 | 5.095 | 5.121 |
|  | 216 | 1 | 1 | 0.234 | 4.268 | 4.288 |
|  | 221 | 1 | 1 | 0.289 | 3.459 | 3.474 |
|  | 210 | 1 | 1 | 0.090 | 11.158 | 11.222 |
|  | 229 | 1 | 1 | 0.130 | 7.678 | 7.720 |
|  | 182 | 1 | 1 | 0.000 | 2289.093 | 2303.483 |
|  | 184 | 1 | 2 | 0.388 | 5.158 | 5.210 |
|  | 177 | 1 | 1 | 0.244 | 4.103 | 4.123 |
|  | 214 | 1 | 1 | 0.567 | 1.765 | 1.770 |
|  | 166 | 1 | 1 | 0.409 | 2.444 | 2.453 |
|  | 213 | 1 | 3 | 0.531 | 5.652 | 5.741 |
|  | 171 | 1 | 2 | 0.218 | 9.175 | 9.278 |
|  | 228 | 1 | 1 | 0.222 | 4.500 | 4.522 |
|  | 183 | 1 | 2 | 0.453 | 4.415 | 4.458 |
|  | 237 | 1 | 1 | 0.363 | 2.755 | 2.766 |
|  | 146 | 1 | 1 | 0.419 | 2.386 | 2.395 |
|  | 209 | 1 | 2 | 0.193 | 10.374 | 10.493 |
| Javiana | 901 | 1 | 2 | 0.191 | 10.462 | 10.623 |
|  | 857 | 1 | 1 | 0.192 | 5.202 | 5.238 |
|  | 875 | 1 | 1 | 0.261 | 3.837 | 3.861 |
|  | 899 | 1 | 1 | 0.129 | 7.779 | 7.837 |
|  | 889 | 1 | 2 | 0.232 | 8.622 | 8.752 |
|  | 902 | 1 | 1 | 0.158 | 6.315 | 6.360 |
|  | 897 | 1 | 1 | 0.165 | 6.076 | 6.119 |
|  | 893 | 1 | 2 | 0.244 | 8.188 | 8.311 |
|  | 870 | 1 | 2 | 0.171 | 11.681 | 11.863 |
|  | 884 | 1 | 2 | 0.014 | 143.560 | 145.997 |
|  | 876 | 1 | 2 | 0.251 | 7.982 | 8.102 |
|  | 881 | 1 | 1 | 0.213 | 4.684 | 4.716 |
|  | 895 | 1 | 1 | 0.149 | 6.721 | 6.769 |
|  | 894 | 1 | 1 | 0.320 | 3.129 | 3.148 |
|  | 887 | 1 | 1 | 0.364 | 2.749 | 2.764 |
|  | 864 | 1 | 1 | 0.198 | 5.053 | 5.087 |
|  | 880 | 1 | 1 | 0.106 | 9.406 | 9.477 |
|  | 865 | 1 | 1 | 0.331 | 3.018 | 3.035 |
|  | 868 | 1 | 1 | 0.062 | 16.258 | 16.387 |
|  | 882 | 1 | 2 | 0.195 | 10.280 | 10.439 |
|  | 888 | 1 | 1 | 0.083 | 12.068 | 12.162 |
|  | 912 | 1 | 2 | 0.349 | 5.736 | 5.817 |
|  | 869 | 1 | 1 | 0.039 | 25.693 | 25.902 |
|  | 908 | 1 | 2 | 0.085 | 23.594 | 23.980 |
|  | 915 | 1 | 1 | 0.100 | 9.990 | 10.066 |
|  | 840 | 1 | 1 | 0.032 | 30.852 | 31.105 |
|  | 790 | 1 | 1 | 0.297 | 3.366 | 3.386 |
|  | 829 | 1 | 1 | 0.110 | 9.092 | 9.160 |
|  | 793 | 1 | 2 | 0.152 | 13.123 | 13.331 |
| Sandiego | 227 | 1 | 1 | 0.200 | 4.993 | 5.026 |
|  | 209 | 1 | 1 | 0.146 | 6.859 | 6.908 |
|  | 249 | 1 | 1 | 0.360 | 2.777 | 2.792 |
|  | 214 | 1 | 1 | 0.428 | 2.334 | 2.345 |
|  | 251 | 1 | 1 | 0.281 | 3.556 | 3.578 |
|  | 241 | 1 | 3 | 0.308 | 9.732 | 9.954 |
|  | 215 | 1 | 8 | 0.384 | 20.833 | 22.237 |
|  | 217 | 1 | 1 | 0.369 | 2.710 | 2.724 |
|  | 268 | 1 | 3 | 0.395 | 7.585 | 7.753 |
|  | 246 | 1 | 2 | 0.321 | 6.239 | 6.327 |
|  | 208 | 1 | 1 | 0.284 | 3.515 | 3.536 |
|  | 247 | 1 | 1 | 0.232 | 4.307 | 4.334 |
|  | 235 | 1 | 1 | 0.288 | 3.470 | 3.491 |
|  | 267 | 1 | 1 | 0.216 | 4.620 | 4.650 |
|  | 213 | 1 | 1 | 0.401 | 2.491 | 2.504 |
|  | 212 | 1 | 2 | 0.403 | 4.960 | 5.027 |
| I 4,[5],12:i:- | 208 | 1 | 2 | 0.082 | 24.266 | 25.676 |
|  | 226 | 1 | 1 | 0.051 | 19.679 | 20.229 |
|  | 218 | 1 | 1 | 0.048 | 20.716 | 21.296 |
|  | 207 | 1 | 1 | 0.084 | 11.883 | 12.203 |
|  | 244 | 1 | 1 | 0.093 | 10.764 | 11.051 |
|  | 215 | 1 | 1 | 0.111 | 8.989 | 9.224 |
|  | 157 | 1 | 1 | 0.089 | 11.282 | 11.584 |
|  | 213 | 1 | 1 | 0.116 | 8.600 | 8.823 |
|  | 180 | 1 | 1 | 0.063 | 15.997 | 16.438 |
|  | 189 | 1 | 1 | 0.075 | 13.390 | 13.755 |
|  | 271 | 1 | 1 | 0.076 | 13.187 | 13.546 |
|  | 178 | 1 | 1 | 0.085 | 11.748 | 12.064 |
|  | 233 | 1 | 1 | 0.030 | 33.177 | 34.123 |
|  | 185 | 1 | 1 | 0.051 | 19.465 | 20.009 |
|  | 272 | 1 | 1 | 0.048 | 21.011 | 21.599 |
|  | 155 | 1 | 1 | 0.086 | 11.680 | 11.994 |
|  | 191 | 1 | 1 | 0.055 | 18.309 | 18.818 |
|  | 197 | 1 | 1 | 0.026 | 37.745 | 38.825 |
|  | 290 | 1 | 1 | 0.031 | 31.768 | 32.673 |

Supplementary Table S2. Local relative risks of zip code areas within the detected significant space-time clusters of illnesses with major *Salmonella* serotypes in Florida, 2017-2018

| Serotype | Location ID | Cluster | Local obs # | Local exp # | Local O/E | Local RR |
| --- | --- | --- | --- | --- | --- | --- |
| Enteritidis | 576 | 1 | 3 | 0.006 | 524.973 | 534.274 |
|  | 574 | 1 | 1 | 0.008 | 119.247 | 119.939 |
|  | 572 | 1 | 1 | 0.008 | 133.282 | 134.056 |
|  | 575 | 1 | 1 | 0.010 | 97.437 | 98.001 |
|  | 571 | 1 | 1 | 0.003 | 300.208 | 301.958 |
|  | 541 | 1 | 1 | 0.023 | 44.263 | 44.516 |
|  | 79 | 2 | 1 | 0.034 | 29.041 | 29.205 |
|  | 86 | 2 | 1 | 0.019 | 53.608 | 53.916 |
|  | 116 | 2 | 1 | 0.012 | 84.885 | 85.376 |
|  | 72 | 2 | 1 | 0.027 | 36.883 | 37.093 |
|  | 11 | 2 | 1 | 0.007 | 134.507 | 135.288 |
|  | 37 | 2 | 1 | 0.019 | 52.432 | 52.733 |
|  | 129 | 2 | 1 | 0.026 | 38.426 | 38.645 |
|  | 31 | 2 | 1 | 0.018 | 55.060 | 55.377 |
|  | 50 | 2 | 1 | 0.033 | 29.903 | 30.072 |
|  | 52 | 2 | 1 | 0.047 | 21.482 | 21.602 |
|  | 54 | 2 | 1 | 0.018 | 55.968 | 56.290 |
|  | 49 | 2 | 1 | 0.007 | 137.710 | 138.510 |
|  | 524 | 2 | 1 | 0.048 | 21.039 | 21.156 |
|  | 519 | 2 | 1 | 0.057 | 17.430 | 17.526 |
|  | 622 | 2 | 1 | 0.024 | 41.321 | 41.557 |
|  | 543 | 2 | 1 | 0.029 | 34.333 | 34.528 |
|  | 620 | 2 | 1 | 0.026 | 38.589 | 38.808 |
|  | 531 | 2 | 1 | 0.039 | 25.322 | 25.464 |
|  | 301 | 2 | 1 | 0.062 | 16.117 | 16.205 |
|  | 538 | 2 | 3 | 0.048 | 62.176 | 63.262 |
|  | 638 | 2 | 1 | 0.040 | 25.214 | 25.355 |
|  | 324 | 2 | 2 | 0.032 | 62.912 | 63.641 |
|  | 511 | 2 | 1 | 0.048 | 20.656 | 20.771 |
|  | 218 | 2 | 2 | 0.030 | 67.055 | 67.832 |
|  | 434 | 3 | 1 | 0.014 | 69.030 | 69.428 |
|  | 407 | 3 | 1 | 0.016 | 62.747 | 63.108 |
|  | 405 | 3 | 1 | 0.008 | 130.797 | 131.556 |
|  | 380 | 3 | 1 | 0.034 | 28.995 | 29.159 |
|  | 831 | 3 | 1 | 0.014 | 72.072 | 72.488 |
|  | 823 | 3 | 1 | 0.033 | 30.521 | 30.694 |
|  | 604 | 3 | 1 | 0.009 | 116.241 | 116.915 |
|  | 369 | 3 | 1 | 0.040 | 24.971 | 25.111 |
|  | 610 | 3 | 1 | 0.014 | 72.521 | 72.940 |
|  | 882 | 3 | 1 | 0.035 | 28.285 | 28.445 |
|  | 911 | 3 | 1 | 0.035 | 28.225 | 28.384 |
|  | 745 | 3 | 1 | 0.014 | 73.551 | 73.975 |
|  | 362 | 3 | 1 | 0.027 | 37.107 | 37.319 |
|  | 881 | 3 | 1 | 0.039 | 25.777 | 25.922 |
|  | 738 | 3 | 1 | 0.021 | 48.723 | 49.003 |
|  | 587 | 3 | 2 | 0.019 | 106.986 | 108.233 |
|  | 890 | 3 | 1 | 0.033 | 30.343 | 30.515 |
|  | 889 | 3 | 1 | 0.042 | 23.722 | 23.855 |
|  | 891 | 3 | 1 | 0.035 | 28.451 | 28.611 |
| Newport | 591 | 1 | 2 | 0.006 | 326.896 | 331.021 |
|  | 850 | 1 | 1 | 0.007 | 139.128 | 139.997 |
|  | 854 | 1 | 1 | 0.010 | 103.391 | 104.035 |
|  | 567 | 1 | 1 | 0.026 | 37.813 | 38.045 |
|  | 561 | 1 | 1 | 0.015 | 64.615 | 65.015 |
|  | 857 | 1 | 1 | 0.022 | 46.414 | 46.700 |
|  | 856 | 1 | 1 | 0.003 | 322.542 | 324.564 |
|  | 884 | 1 | 1 | 0.002 | 640.393 | 644.414 |
|  | 573 | 1 | 1 | 0.014 | 71.738 | 72.183 |
|  | 892 | 1 | 1 | 0.026 | 38.740 | 38.977 |
|  | 657 | 1 | 3 | 0.036 | 82.635 | 84.195 |
|  | 538 | 1 | 2 | 0.030 | 67.204 | 68.042 |
|  | 775 | 1 | 1 | 0.015 | 67.801 | 68.222 |
|  | 908 | 1 | 1 | 0.010 | 105.248 | 105.903 |
|  | 177 | 2 | 1 | 0.030 | 32.944 | 33.145 |
|  | 179 | 2 | 2 | 0.074 | 26.933 | 27.261 |
|  | 212 | 2 | 1 | 0.066 | 15.058 | 15.146 |
|  | 174 | 2 | 2 | 0.045 | 44.305 | 44.853 |
|  | 155 | 2 | 1 | 0.049 | 20.544 | 20.667 |
|  | 184 | 2 | 1 | 0.048 | 20.704 | 20.828 |
|  | 183 | 2 | 1 | 0.056 | 17.722 | 17.827 |
|  | 153 | 2 | 1 | 0.070 | 14.326 | 14.409 |
|  | 194 | 2 | 1 | 0.029 | 34.403 | 34.613 |
|  | 216 | 2 | 1 | 0.029 | 34.264 | 34.473 |
|  | 213 | 2 | 1 | 0.066 | 15.126 | 15.215 |
|  | 147 | 2 | 1 | 0.056 | 17.906 | 18.012 |
|  | 209 | 2 | 1 | 0.024 | 41.645 | 41.900 |
|  | 236 | 2 | 1 | 0.043 | 23.431 | 23.572 |
| Javiana | 895 | 1 | 1 | 0.006 | 163.922 | 165.303 |
|  | 897 | 1 | 1 | 0.007 | 148.194 | 149.441 |
|  | 881 | 1 | 1 | 0.009 | 114.257 | 115.217 |
|  | 882 | 1 | 1 | 0.008 | 125.374 | 126.428 |
|  | 912 | 1 | 1 | 0.014 | 69.949 | 70.534 |
|  | 887 | 1 | 1 | 0.015 | 67.051 | 67.610 |
|  | 868 | 1 | 1 | 0.003 | 396.535 | 399.887 |
|  | 908 | 1 | 2 | 0.003 | 575.473 | 585.293 |
|  | 857 | 1 | 1 | 0.008 | 126.892 | 127.959 |
|  | 901 | 1 | 1 | 0.008 | 127.585 | 128.658 |
|  | 38 | 2 | 1 | 0.012 | 85.629 | 86.346 |
|  | 41 | 2 | 1 | 0.028 | 36.053 | 36.350 |
|  | 42 | 2 | 1 | 0.024 | 41.875 | 42.222 |
|  | 487 | 2 | 1 | 0.019 | 52.675 | 53.113 |
|  | 85 | 2 | 1 | 0.021 | 47.725 | 48.121 |
|  | 481 | 2 | 1 | 0.019 | 51.718 | 52.148 |
|  | 102 | 2 | 1 | 0.016 | 62.989 | 63.515 |
|  | 477 | 2 | 1 | 0.021 | 46.887 | 47.276 |
|  | 105 | 2 | 1 | 0.010 | 95.669 | 96.471 |
|  | 494 | 2 | 1 | 0.013 | 76.300 | 76.938 |
|  | 538 | 2 | 3 | 0.033 | 90.855 | 93.179 |
|  | 107 | 2 | 2 | 0.030 | 66.301 | 67.417 |
| Sandiego | 215 | 1 | 5 | 0.048 | 103.398 | 107.812 |
|  | 264 | 2 | 1 | 0.006 | 164.123 | 165.483 |
|  | 268 | 2 | 1 | 0.033 | 30.283 | 30.527 |
|  | 257 | 2 | 1 | 0.013 | 77.301 | 77.937 |
|  | 261 | 2 | 1 | 0.014 | 71.903 | 72.494 |
|  | 246 | 2 | 2 | 0.027 | 74.727 | 75.966 |
|  | 231 | 2 | 1 | 0.008 | 120.789 | 121.787 |
|  | 858 | 3 | 1 | 0.002 | 406.902 | 410.284 |
|  | 893 | 3 | 1 | 0.021 | 48.299 | 48.693 |
|  | 881 | 3 | 1 | 0.018 | 55.263 | 55.715 |
|  | 879 | 3 | 2 | 0.014 | 139.651 | 141.981 |
|  | 819 | 3 | 1 | 0.010 | 102.584 | 103.430 |
|  | 771 | 3 | 1 | 0.015 | 64.730 | 65.261 |
|  | 894 | 3 | 1 | 0.027 | 36.919 | 37.219 |
|  | 888 | 3 | 1 | 0.007 | 142.367 | 143.545 |
|  | 821 | 3 | 1 | 0.012 | 82.811 | 83.493 |
| Braenderup | 678 | 1 | 1 | 0.009 | 106.546 | 107.773 |
|  | 931 | 1 | 1 | 0.005 | 191.328 | 193.541 |
|  | 929 | 1 | 1 | 0.001 | 698.818 | 706.932 |
|  | 722 | 1 | 1 | 0.016 | 61.710 | 62.416 |
|  | 735 | 1 | 1 | 0.004 | 265.239 | 268.311 |
|  | 617 | 1 | 2 | 0.010 | 191.802 | 196.291 |
|  | 730 | 1 | 1 | 0.007 | 135.835 | 137.403 |
|  | 798 | 1 | 1 | 0.008 | 127.909 | 129.385 |
|  | 332 | 1 | 1 | 0.002 | 478.817 | 484.373 |
|  | 572 | 1 | 1 | 0.008 | 129.590 | 131.085 |
|  | 574 | 1 | 1 | 0.009 | 115.944 | 117.281 |
| Typhimurium | 414 | 1 | 1 | 0.002 | 596.234 | 604.066 |
|  | 390 | 1 | 1 | 0.005 | 186.528 | 188.969 |
|  | 380 | 1 | 1 | 0.010 | 97.684 | 98.957 |
|  | 740 | 1 | 1 | 0.006 | 154.647 | 156.669 |
|  | 371 | 1 | 1 | 0.001 | 1248.172 | 1264.582 |
|  | 912 | 1 | 1 | 0.019 | 53.166 | 53.852 |
|  | 548 | 1 | 1 | 0.008 | 122.682 | 124.283 |
|  | 857 | 1 | 1 | 0.010 | 96.446 | 97.702 |
|  | 362 | 1 | 1 | 0.008 | 125.014 | 126.645 |
|  | 900 | 1 | 1 | 0.010 | 103.458 | 104.806 |
|  | 498 | 1 | 1 | 0.006 | 169.330 | 171.545 |
|  | 518 | 1 | 1 | 0.010 | 104.834 | 106.200 |
|  | 497 | 1 | 1 | 0.006 | 175.338 | 177.632 |
| I 4,[5],12:i:- | 226 | 1 | 1 | 0.006 | 158.239 | 162.863 |
|  | 218 | 1 | 1 | 0.006 | 166.574 | 171.443 |
|  | 215 | 1 | 1 | 0.014 | 72.278 | 74.374 |
|  | 207 | 1 | 1 | 0.010 | 95.547 | 98.328 |
|  | 244 | 1 | 1 | 0.012 | 86.551 | 89.067 |
|  | 213 | 1 | 1 | 0.014 | 69.148 | 71.153 |
|  | 180 | 2 | 1 | 0.008 | 128.631 | 132.384 |
|  | 185 | 2 | 1 | 0.006 | 156.520 | 161.094 |
|  | 191 | 2 | 1 | 0.007 | 147.220 | 151.521 |
|  | 197 | 2 | 1 | 0.003 | 303.502 | 312.399 |
|  | 189 | 2 | 1 | 0.009 | 107.670 | 110.807 |
